# Understanding and benchmarking health service achievement of policy goals for chronic disease

**DOI:** 10.1186/1472-6963-12-343

**Published:** 2012-09-29

**Authors:** Erica Bell, Bastian Seidel

**Affiliations:** 1 University Department of Rural Health, University of Tasmania, Burnie, Tasmania, Australia; 2 Discipline of General Practice, University of Tasmania, Burnie, Tasmania, Australia

## Abstract

**Background:**

Key challenges in benchmarking health service achievement of policy goals in areas such as chronic disease are: 1) developing indicators and understanding how policy goals might work as indicators of service performance; 2) developing methods for economically collecting and reporting stakeholder perceptions; 3) combining and sharing data about the performance of organizations; 4) interpreting outcome measures; 5) obtaining actionable benchmarking information. This study aimed to explore how a new Boolean-based small-*N* method from the social sciences—Qualitative Comparative Analysis or QCA—could contribute to meeting these internationally shared challenges.

**Methods:**

A ‘multi-value QCA’ (MVQCA) analysis was conducted of data from 24 senior staff at 17 randomly selected services for chronic disease, who provided perceptions of 1) whether government health services were improving their achievement of a set of statewide policy goals for chronic disease and 2) the efficacy of state health office actions in influencing this improvement. The analysis produced summaries of configurations of perceived service improvements.

**Results:**

Most respondents observed improvements in most areas but uniformly good improvements across services were not perceived as happening (regardless of whether respondents identified a state health office contribution to that improvement). The sentinel policy goal of using evidence to develop service practice was not achieved at all in four services and appears to be reliant on other kinds of service improvements happening.

**Conclusions:**

The QCA method suggested theoretically plausible findings and an approach that with further development could help meet the five benchmarking challenges. In particular, it suggests that achievement of one policy goal may be reliant on achievement of another goal in complex ways that the literature has not yet fully accommodated but which could help prioritize policy goals. The weaknesses of QCA can be found wherever traditional big-*N* statistical methods are needed and possible, and in its more complex and therefore difficult to empirically validate findings. It should be considered a potentially valuable adjunct method for benchmarking complex health policy goals such as those for chronic disease.

## Background

Wide variation in achievement of both voluntary and mandatory quality improvement policies and strategies in different organizations is a difficult management issue for policy-makers [1]. How should the achievement of policy goals by health services be understood and benchmarked? The weaknesses of benchmarking and productivity monitoring systems for organizational performance and improvement are many and well documented in the literature [2]—there are over 4,000 references in PUBMED involving the terms ‘benchmarking’ and ‘quality’, almost all published since 2000. Yet there is general agreement that methods for comparative measurement of care quality and its improvement are not yet well-developed, including for measuring policy frameworks [3-8]. Detailed discussion of the benchmarking literature is outside the scope of this paper, however, five key challenges in benchmarking are briefly highlighted here to help explain the aims of our study.

A key challenge in organizational benchmarking is identification of indicators. The Institute of Medicine has defined quality in terms of safe, effective, appropriate, patient-centered, timely, efficient and equitable care [9,10]. Since the 1978 *Science* paper by Donabedian on the subject, indicators have often been described in terms of healthcare structure, process or outcomes [11]. The literature on benchmarking emphasizes indicators that can help improve clinical practice as well as the organizational and wider frameworks in which practitioners operate [12]. Yet there is little consensus in the benchmarking literature about the measurement of performance of healthcare organizations [13]. This may arise from the diversity of areas that have been ‘benchmarked’, which range from clinical practice to electronic medical records systems [14] to service quality in discrete disease areas [15]. Clinical guidelines set quality benchmarks for practice developed by organizations such as the UK’s National Institute for Clinical Excellence (NICE) and are the subject of a large body of literature—over 4,900 PUBMED references (more evenly spread over the last two decades than the lately escalating volume of benchmarking literature). However, the clinical standards literature, like the NICE guidelines, is focused on discrete disease areas rather than broader systemic policy issues [16-20].

A growing literature exists in assessing whole-of-service and whole-of-system achievement of specific policy goals in different kinds of health services [21-24]. For example, achievement of the health policy goal of engagement of HIV patients in a continuum of care model in the UK has been measured using a socio-ecological perspective to understand the interplay of individual, community, and health system and policy-level factors that influence the way that engagement in HIV care operates [25]. However, to date little is known about how health service policy goals work to help improve services across a health system and whether and how policy goals themselves could work as indicators of performance. Do local area health service mangers and practitioners recognize them as meaningfully connected to state health office actions? Are they able to see real improvements in services in terms of state health goals? This study aimed to explore the answers to such questions.

The second challenge is about who provides the performance data relevant to chosen indicators. The success of benchmarking relies on whether and how patients, clinicians, managers, and other health system stakeholders are involved in data collection and reporting for benchmarking [12]. Administrative and/or clinical data are often used in benchmarking exercises, however, the former may not have been collected for the purposes of benchmarking [10] and the latter may often leave out key dimensions shaping quality. Other empirical sources include population health data and patient data held in registries.

While data from service stakeholders can be different from more objective measures, such differences can themselves help raise questions about the quality and accuracy of these objective measures [25-29]. This study also aimed to make a contribution to methods for economically collecting and reporting the service performance perceptions of two key stakeholders—practitioners and service managers.

The analysis of benchmarking data represents a third key challenge. Understanding real variations (and trends) in organizational performance is a task that requires an effort of not only data collection but also shared analysis including those directly involved in the day-to-day management of those organizations [30]. In ‘best practice’ benchmarking exercises, the features of high-performing organizations are identified in a highly iterative process involving both external benchmarks and internal goals that is fully integrated into organizational operation and hopefully policy-making [31,32]. In reality though, many factors from pragmatic time constraints to ethics requirements for confidentiality can limit the extent to which lesson sharing about higher and lower performing organizations is possible. This study also aimed to explore feasible ways of combining and sharing data about the performance of organizations across a system that helps create dialogue between services and policy-makers.

The interpretation of outcome measures is a fourth challenge. Benchmarking indicators are shaped by the quality of the (implicit or explicit) classification systems that are used to build and interpret them in a context of what is often complex health phenomena [33]. A key question is about precisely what aspects of clinical and organizational practice are at work, in what ways, to shape the outcomes observed [34]. It is known that going beyond measuring simple routines requires more complex indicators, however, the greater the complexity of the indicators used, the greater the difficulties of interpretation involved [35,36].

Sophisticated benchmarking approaches focus on how systems work, including how the social dimensions of organizations such as hospitals operate [37]. Accordingly, the aim of understanding health service achievement of policy goals should be less about developing league tables of these organizations using narrow datasets [38] and more about understanding how services operate to achieve those goals. This study aimed to help meet this fourth key challenge—of interpreting outcome measures (in this case policy goals) in complexity-oriented ways. It aimed to explore a possible new method for considering the ways in which service improvement occurs combinatorially. That is, we aimed to contribute answers to the question of how service improvements work one in relation to another within a system. Are some kinds of improvements necessary before other kinds of improvements are possible?

The use of benchmarks represents a fifth critical challenge [39,40]. Benchmarks are purported to be useful for driving improvement by helping identify interventions that are working; facilitating accountability to stakeholders through reporting mechanisms; providing a basis for the development of incentives systems [41]. However, simplistic approaches to using benchmarking information, such as performance targets [42] or organization scorecards [43] or public reporting [44] have had uneven results. There are reports that practitioners, service managers, policy-makers and patients do not use performance data because they have not been involved properly in the benchmarking process or because the data has relevance problems or is difficult to understand [45-48]. This study aimed to contribute to the challenge of obtaining benchmarking information that is actionable and targets end users (in this case policy-makers) [10]. In so doing it considered a particularly complex challenge for policy-makers— health service achievement of state policy goals for chronic disease across the system. It aimed to explore the usefulness of a possible new method from the social sciences—Qualitative Comparative Analysis or QCA—for developing understandings of the achievement of policy goals.

## Methods

This study used a QCA-based analysis of service manager and practitioner perceptions of

• whether government health services were improving their achievement of a set of statewide policy goals for chronic disease and

• the efficacy of state health office actions in influencing this improvement.

Thus, the method aimed to target not only improvements in performance of policy goals by health services, but also the contribution of the state health office to that improvement. This represents a double-edged conceptualization of performance consistent with the idea that the performance of any service entity cannot be understood abstracted from its larger operating system.

Accordingly, the specific research questions were twofold. The first question was ‘What differences can be found between those who identified a state health contribution and those who did not, in terms of the nature and extent of service improvement?’ Answering this question required treating the presence of a perceived state health office contribution to improvement as an outcome measure. The second research question was ‘Given that evidence-based practice is a sentinel policy goal, what kinds of perceived improvements might be necessary or sufficient to achieve this particular improvement i.e. are certain kinds of improvements linked to this critical improvement?’ Answering this question required treating the presence of at least some perceived improvement in evidence-based practice as an outcome measure.

Table 1 provides the details of the survey instrument. Each of the eight questions corresponds (in order) to the eight ‘in principle’ operating goals for health services in the state of Tasmania, Australia, provided to the consultant by the state health office (the Department of Health and Human Services or DHHS):

1. Adopt a population health approach and address health inequity

2. Adopt a person-centered approach

3. Work in health promoting ways

4. Integrate self-management into chronic disease prevention and management

5. Adopt evidence-informed practice and policy-making

6. Facilitate coordinated and integrated multidisciplinary care

7. Strengthen partnerships and collaborations

8. Commit to surveillance, monitoring, evaluation and research.

**Table 1:** QCA chronic disease survey

**Manager**/**practitioner belief about whether improvement is happening**	**COLUMN A**: **Degree of improvement *at your service*** (**circle one only**)	**COLUMN B**: **DHHS actions that havemost influenced your answer**, **e**.**g**., **DHHS** ‘**Chronic disease action framework**’, **DHHS** ‘**Self Management Framework**’, **DHHS** ‘**Health Promotion Framework**’ **etc**. (**leave blank if youcannot name any suchDHHS actions or elsename some other actionthat has most influencedyour answer**).
I believe my service is *getting better* at meeting the needs of those with unequal health outcomes	O = No, not at all	
	1 = Yes, a little better	
	2 = Yes, a great deal better	
	U = Unable to decide.	
I believe my service is *getting better* at focussing on the needs of the whole patient	O = No, not at all	
	1 = Yes, a little better	
	2 = Yes, a great deal better	
	U = Unable to decide.	
I believe my service is *getting better* at health promotion to reduce chronic disease	O = No, not at all	
	1 = Yes, a little better	
	2 = Yes, a great deal better	
	U = Unable to decide.	
I believe my service is *getting better* at also supporting clients to effectively self-manage their chronic conditions	O = No, not at all	
	1 = Yes, a little better	
	2 = Yes, a great deal better	
	U = Unable to decide.	
I believe my service is *getting better* at using evidence in its decision-making practices	O = No, not at all	
	1 = Yes, a little better	
	2 = Yes, a great deal better	
	U = Unable to decide.	
I believe my service is *getting better* at facilitating coordination of care	O = No, not at all	
	1 = Yes, a little better	
	2 = Yes, a great deal better	
	U = Unable to decide.	
I believe my service is *building better* links with other practitioners and healthcare services and agencies who can make a difference to healthcare	O = No, not at all	
	1 = Yes, a little better	
	2 = Yes, a great deal better	
	U = Unable to decide.	
I believe my service is *getting better* at collecting and using information about itself to help improve quality of care	O = No, not at all	
	1 = Yes, a little better	
	2 = Yes, a great deal better	
	U = Unable to decide.	

The study included services operating in Tasmania that have involvement in chronic disease prevention or treatment. That is, it did not include all state government services, just a random sample of 20 services (using SPSS) taken from a larger list of 137 services held by the state health office which included all state health services directed at people living with, or at risk of, chronic conditions. Surveys were distributed by the state health office, as required by ethics approval processes, and directed at two senior staff (whether primarily managers or clinicians) in each service who were in a position to take a whole-of-service improvement perspective. The state health office in Tasmania provides or funds others to provide a wide range of services across the broad spectrum of healthcare within the framework of Australia’s public health system. The staff surveyed were local public health employees who were also care providers (actively involved in directly managing or delivering care as senior practitioners) in the following kinds of health services: key units within hospitals; chronic disease prevention services, community health centres; specialist chronic disease treatment services, including mental health services.

The responses to the survey were either in writing or by telephone interview, as nominated by participants. Practitioners and service managers were encouraged to use their professional judgment to interpret the policy goal questions in ways appropriate to their services, on the survey form and in telephone prompts. Respondents included 17 health service managers and 7 clinicians in 17 services (not the balanced number of managers and clinicians as originally hoped).

The data for the outcome relevant to efficacy of state health office actions (column B in Table 1) were coded in the following way: ‘0’ for no DHHS action identified and ‘1’ for a DHHS action identified for any of the survey items. Those who indicated they were unable to decide were coded as not observing an improvement (i.e. for the purpose of this study, being definitive about not seeing an improvement is the same as saying you are undecided whether you can see an improvement). Thus, the survey questions involved three grades of responses (0,1,2) except for the outcome of identifying a state health office action which involved two grades of responses (0,1).

The key concepts in the QCA method are described in a more technical appendix provided at the end of this paper, with references. This section describes the key practical steps in the QCA analysis conducted for this study using simplified non technical language.

Step 1. Enter the data onto the software spreadsheet. The data from the survey were entered on a data spreadsheet in the QCA software TOSMANA, with each row an instance of the survey form, and each column a variable (or case condition and outcome) of interest. The values (0,1,2) for the survey questions were entered in this step, except where an outcome was being considered which involved entering two values (0,2).

Step 2. Run the software to produce an initial tabular summary of the cases. In this step the researcher obtained a summary of observed conditions of cases for the outcomes of interest i.e. a summary of *the combinations* of variables *defining individual cases*. Cases with the same combination of conditions and the same outcome were summarized in a single line (of notation) by the software. Cases with different combinations of conditions and the same outcome were summarized in different lines of notation by the software. Therefore, each line of the tabular summary produced in step 2 represented a unique combination of variables entered in step 1.

Step 3. Examine odd or contradictory features of the software output from step 2. In this stage the researcher examined the summary of the observed dataset produced in step 2, by going back to the original data and considering it in the light of the relevant research literature (described in the background to this paper). That is, the distinct combinations of variables summarizing individual cases provided by step 2 were examined by asking questions such as ‘Are there any unsupportable contradictions in these summaries of case conditions and outcomes?’ For example, cases with the same outcome but different combinations of conditions were reconsidered in the light of the original survey evidence to see if there have been any errors in data entry or in interpretation of the survey forms etc.and data should be revised accordingly. Therefore, this step involved iterative checking and re-checking of the software output.

Step 4. Use the QCA software to minimize or summaries all the unique combinations of case conditions and outcomes from steps 2 and 3. This step involved producing ‘equations’ that summaries the table produced by the software in step 2 and checked in step 3. That is, the software was used to further summaries all the unique combinations of case conditions and outcomes recorded in that table. To do this ‘minimization’ or summarizing the software uses an algorithm that involves comparing every unique combination identified for the outcome specified. The algorithm will identify and remove variables that are both present *and* absent in combinations or configurations for an outcome that are otherwise alike in every other respect. For example, if the postvan comes every Tuesday and every Thursday when the sky is blue, regardless of whether I sit on the front steps of my house or not, we could assume that the last observation is irrelevant to the outcome being observed. Such a variable could not be described as a causal factor for that outcome which has occurred regardless of the presence of that variable. The algorithm removes such irrelevant variables to produce a simpler, combined expression describing possible causal factors for an outcome.

Step 5. Interpret equations summarizing observed cases. In this step the researcher describes, as seen in the findings section of this paper, the different ‘equations’ or summaries delivered by Step 4. One set of equations is produced for cases where the outcome is present, and another set of equations for cases where the outcome is absent. The researcher describes what all the equations suggest as a whole about conditions (possible causal variables) that may be important to a particular outcome. For example, if there is one equation for an outcome and that equation has only one variable left after the ‘minimization’ algorithm in step 4 has been performed, this suggests that the variable may be sufficient on its own to produce the outcome. On the other hand, if there is one equation with a combination or string of variables for an outcome this suggests that the different variables in that combination are necessary to deliver that outcome i.e. each variable in that combination interacts with another. However, if the equations produced suggest that different combinations of conditions could lead to the same outcome then it is likely that complex causality is operating i.e. no one condition identified by the survey is necessary or sufficient for that outcome.

Step 6. Use the software to consider all missing combinations of case conditions. All the previous steps have involved summarizing what could be said about observed or real cases in the dataset. In this step, all the possible combinations of case conditions (all possible missing cases) are considered and added to the observed dataset. That is, the QCA software automatically generates all the possible combinations of case conditions for the outcomes of interest as if every single possible missing case existed. Previous steps 2-5 are then repeated to ‘minimize’ these missing cases and arrive at understandings of equations summarizing what case conditions are *in theory* necessary or sufficient to a particular outcome.

Accordingly, the QCA analyses that follow attempt to summarise what is necessary and sufficient (or neither) for the outcomes of interest 1) whether or not the practitioner or service manager perceives a state health contribution; 2)whether or not there is improvement in the sentinel area of evidence-based practice. The analyses also attempt to explore what conditions are theoretically necessary or sufficient to these outcomes when all possible missing cases are included (given that all possible permutations of views are theoretically possible i.e. we can’t eliminate some on the basis of known evidence or theory about such views in Tasmania).

(This research has received ethical approval from The Human Research Ethics Committee Tasmania network Ref. No: H0011753).

## Results

### A simple ‘non QCA map’ of survey responses

Table 2 provides the frequencies for the different responses to the survey questions (as the total number of respondents is 24 for each policy goal area, the numbers involved are too small to provide percentages). It suggests that the majority of responses in each and every policy goal area included at least some and often a great deal of observed improvement i.e. ranged from ‘a little better’ to ‘a great deal better’. Respondents who advised they saw no improvement were therefore in the minority of cases. The policy goal area that relates to services getting better at facilitating coordination of care was the one where respondents were notably unable to decide.

**Table 2:** **Frequency table for perceiveddegrees of improvement underkey chronic disease policygoals**

**Degree of improvement**	**inequity**	**whole patient**	**health promotion**	**self**-**manage**	**using evidence**	**coordination**	**links**	**improve quality**
Blank	1	1	0	1	1	0	0	0
Unable to decide	1	0	3	0	3	11	0	1
No, not at all	1	3	2	2	3	0	1	3
Yes, a little better	14	14	9	13	7	12	14	11
Yes, a great deal better	7	6	9	8	10	11	9	9

Fourteen of the 24 respondents identified a state health office action that had shaped their view of their service’s improvement. Included in these 14 is one clinician respondent who advised there were ‘system barriers’ to improvement but did not identify a specific state health office action (while identifying no to a little improvement in his/her service under the different policy goals).

Of those 10 who did not identify a state health office action, one commented that it was not possible to make any assessment as there was no shared idea of how to understand service improvement; another claimed that services had not improved because they were ‘good already’ (most left the form blank as requested).

### A QCA analysis of state health office contributions and service improvement

Example 1 provides sample combinations or configurations for observed cases with the outcome where a state health office action had been identified as relevant to degree of service improvement i.e. it is extracted from an analysis of 14 of the 24 respondents. In this and the boxes that follow, services are given an ID number 1 to 17 with ‘M’ for manager and ‘C’ for clinician respondents; asterisk * means ‘AND’ while plus + means ‘OR’. The observed cases (involving 13 configurations because two configurations from different services are the same) suggest that no to great service improvements were identified by this group in quite different areas. That is, among those who identified at least one state health office action leading to improvements, there was no pattern of uniform improvement across services observed in any specific area of the statewide policy goals for chronic disease. Yet most respondents observed improvements in most areas; only 6 respondents observed no improvements in one or more areas.

The observed configurations suggest that, even among those inclined to see the state health office as having a role in improving health services for chronic disease, it is clear that service improvements are perceived as happening differently across different services.

Example 1: Example configurations (from the observed dataset) for the outcome of having identified a state health office action that has contributed to improvement in the following policy goal areas:

Adopt a population health approach and address health inequity (INEQUITY)

Adopt a person-centred approach (WHOLE PATIENT)

Work in health promoting ways (HEALTH PROM)

Integrate self-management into chronic disease prevention and management (SELF-MANAGE)

Adopt evidence-informed practice and policy-making (EVIDENCE)

Facilitate coordinated and integrated multidisciplinary care (COORD)

Strengthen partnerships and collaborations (LINKS)

Commit to surveillance, monitoring, evaluation and research (QUAL IMPROVE)

Example configuration (where 0 = no improvement; 1 = a little improvement; 2 = a big improvement and a plus sign + means OR):

*For service manager M1 only a little improvement {value = 1} was observed across all policy goal areas in that service i.e. the configuration of responses on the survey form can be summarized as:*

$$\begin{array}{l} \text{INEQUITY}\left\{1\right\}*\text{WHOLE PATIENT}\left\{1\right\}*\text{HEALTH PROM}\left\{1\right\}*\text{SELF-MANAGE}\left\{1\right\}*\\ \text{EVIDENCE}\left\{1\right\}*\text{COORD}\left\{1\right\}*\text{LINKS}\left\{1\right\}*\text{QUAL IMPROVE}\left\{1\right\} \end{array}$$

*For clinician C4 two policy areas (coordination of care and linking with other healthcare providers) were observed to have greatly improved {value = 2}*

$$\begin{array}{l} \text{INEQUITY}\left\{1\right\}*\text{WHOLE PATIENT}\left\{1\right\}*\text{HEALTH PROM}\left\{1\right\}*\text{SELF-MANAGE}\left\{1\right\}*\\ \text{EVIDENCE}\left\{1\right\}*\text{COORD}\left\{2\right\}*\text{LINKS}\left\{2\right\}*\text{QUAL IMPROVE}\left\{1\right\}\; \end{array}$$

However, if every single possible missing perception about service improvement was added to our sample, what would remain true about a hypothetical group of managers and practitioners who see the state health office as having had an impact on their service (of some kind)? The QCA method allows us to add all these missing cases to answer this question. Example 2 summarizes what degree and kinds of perceived service improvements seem to characterize those who see a positive (and in one case possibly negative) role for the state health office. As such, Box 2 offers a single long ‘logical equation’ summarizing perceived improvements in the health system for those who can also see state health office impact of some kind.

Example 2: Logical equation for the outcome of having identified a state health office action that has shaped degree of improvement observed (including all possible missing cases added to observed combinations of survey responses).

Logical equation (where 0 = no improvement; 1 = a little improvement; 2 = a big improvement and a plus sign + means OR):

$$\begin{array}{l} \text{LINKS}\left\{0\right\}+\\ \text{WHOLE}\;\text{PATIENT}\left\{0,2\right\}\text{LINKS}\left\{2\right\}+\\ \text{WHOLE PATIENT}\left\{1\right\}\text{SELF-MANAGE}\left\{2\right\}+\\ \text{WHOLE PATIENT}\left\{1\right\}\text{EVIDENCE}\left\{1\right\}\text{QUAL IMPROVE}\left\{1\right\}+\\ \text{SELF-MANAGE}\left\{1\right\}\text{EVIDENCE}\left\{2\right\}\text{LINKS}\left\{1\right\} \end{array}$$

How should these findings be understood? In QCA terminology, logical equations can be used to contradict or support ‘set theoretic’ claims i.e. claims about how the world works. The logical equation in Box 2 is suggesting what, on the basis of not only these observed data but also all possible missing cases, is theoretically necessary or even sufficient (or neither) for the outcome observed. It can be therefore considered a kind of theoretical explanation of that outcome.

Thus, Example 2 shows that those who indicate they believe that the state health office has had some impact on their service also, in theory, variously indicate the following conditions for this outcome:

• there has been no improvement in building better links with other practitioners/services (i.e. retrieval of the relevant survey form clarified that this part of the logical equation is explaining the case where health system actions were seen as being negative in impact) *OR*

• a big improvement in links to other services/practitioners, connected to *both* no or large perceived improvements in whole-of-patient care (this suggests that no and large improvements in whole-of-patient care may be shaped by quite different factors that nonetheless are connected to perceived big improvements in links to other services/practitioners) *OR*

• a little improvement in whole-of-patient approaches, connected with a big improvement in patient self-management *OR*

• a little improvement in the areas of whole-of-patient approaches *and* using evidence in decision-making practices, connected to a small improvement in using evidence in quality improvement *OR*

• a big improvement in the use of evidence in decision-making, connected to small improvements in patient self-management *and* small improvements in building links to other services and practitioners.

Example 3 offers example configurations for the outcome of not identifying any state health office actions that have had a role in observed improvements i.e. two examples taken from the 10 out of the 24 respondents who indicated this perception. The observed cases also suggest that no to great improvements were identified by this group in quite different areas. Again, the QCA-based configurations suggested there is no immediately obvious uniform pattern to these responses. That is, those who could not identify a state health office action that had shaped their service were in fact quite able to variously observe a wide range of improvements and lack of improvements in their services.

Example 3: Example configurations for the outcome of not having identified a state health office action that has shaped degree of improvement observed (observed configurations only)

*For service manager M4 no improvement {0} was observed across all policy goal areas except for coordination of care (great improvement or {2}) and links to other services (a little improvement or {1})*

$$\begin{array}{l} \text{INEQUITY\{0\}*WHOLE PATIENT\{0\}*HEALTH PROM\{0\}*SELF-MANAGE\{0\}*}\\ \text{COORD\{2\}*LINKS\{1\}*QUAL IMPROVE\{0\}+} \end{array}$$

*For clinician C7 a little improvement {1} was observed in 5 policy goal areas and great improvement {2} was observed in one policy goal (evidence-based practice) but no improvement {0} was observed in 2 policy goal areas*

$$\begin{array}{l} \text{INEQUITY\{1\}*WHOLE PATIENT\{1\}*HEALTH PROM\{0\}*EVIDENCE\{2\}*}\\ \text{COORD\{1\}}\text{*LINKS\{1\}*QUAL IMPROVE\{1\}} \end{array}$$

Example 4 summarizes, in the form of a ‘logical equation’ produced by the QCA software, what in theory might be true for those who see state health office actions as having had no impact on their service (of any kind), by adding to this limited sample, and then reducing using the QCA TOSMANA software, every single possible missing perception (that could be obtained from this survey form) about service improvement.

Example 4: Logical equation for the outcome of not having identified a state health office action that has shaped degree of improvement observed (including all possible missing cases).

Logical equation:

$$\begin{array}{l} \text{SELF-MANAGE}\left\{0\right\}+\\ \text{WHOLE PATIENT}\left\{0,2\right\}\text{LINKS}\left\{1\right\}+\\ \text{COORD}\left\{1\right\}\text{LINKS}\left\{2\right\}+\\ \text{WHOLE PATIENT}\left\{1\right\}\text{HEALTH PROM}\left\{1\right\}\text{EVIDENCE}\left\{0\right\} \end{array}$$

Example 4 suggests that, in theory, those who cannot identify any state health office action as having an impact on their services (in any policy area) also variously indicate the following conditions for this outcome:

no improvement in the capacity of services to support clients to self-manage *OR*

a small improvement in links to other services, connected to *both* no or large perceived improvements in whole-of-patient care (again, this suggests that no and large improvements in whole-of-patient care are likely shaped by quite different factors) *OR*

a small improvement in coordination of care, connected to a big improvement in service/practitioner links *OR*

a small improvement in both whole-of-patient and health promotion, connected to no improvement in using evidence in decision-making.

### Using QCA to understand interactions between different kinds of service improvements

The foregoing analysis focused on understanding similarities and differences between two groups: those who observed at least one state health office action that had improved their services and those who did not. Of course, a QCA analysis can be used to explore other kinds if phenomena in these data. For example, we can use the QCA method to explore what kinds of service improvements might be linked or conditional on one another in different ways, at least at the indicative level of practitioners and service managers’ experiences. This can be useful information for policy in deciding what to prioritise when trying to achieve a particular policy goal.

The use of evidence to improve the quality of a service might be considered a sentinel outcome that would involve other aspects of services as necessary or even sufficient conditions for its achievement. There were two clinicians and two managers from four different services that advised they had not observed any improvement whatsoever in the collection and use of information to improve quality of care at their service. Were their perceptions of other improvements different from the larger group that saw some improvement in this policy goal? What does the answer to this question tell us about how service improvement might work?

Example 5 presents the logical equations when all missing cases are considered on this matter. The first set of equations suggests that reporting a little to great improvement in evidence-based service practice is a situation that has multiple explanatory pathways. These can variously involve big improvements in other areas such as whole-of-patient care or health promotion or patient self-management or a little improvement in whole of patient care and a little to a great deal of improvement in coordination of care. The existence of all these explanatory pathways suggests a situation of causal complexity.

Example 5 also suggests in two other equations offering different explanatory pathways that reporting no improvement in evidence-based service practice is accompanied by, and may be conditional on, no improvement in coordination of care or whole-of-patient approaches (when the latter is accompanied by no to a little improvement in health promotion *or* no to a little improvement in patient self-management).

Example 5: Logical equations for both outcomes for the item ‘getting better at collecting and using evidence to improve quality of care’ ( i.e. no improvement versus a little to great improvement), including all possible missing cases

*A little to great improvement observed for this item produced three logical equations:*

$$\begin{array}{l} \text{WHOLE PATIENT}\left\{2\right\}+\text{HEALTH PROM}\left\{2\right\}+\text{WHOLE PATIENT}\left\{1\right\}\text{COORD}\left\{1,2\right\}\\ \text{WHOLE PATIENT}\left\{2\right\}+\text{SELF-MANAGE}\left\{2\right\}+\text{WHOLE PATIENT}\left\{1\right\}\text{COORD}\left\{1,2\right\}\\ \text{SELF-MANAGE}\left\{2\right\}+\text{EVIDENCE}\left\{1\right\}+\text{WHOLE PATIENT}\left\{1\right\}\text{COORD}\left\{1,2\right\} \end{array}$$

*No improvement observed for this item produced two logical equations:*

$$\begin{array}{l} \text{COORD}\left\{0\right\}+\text{WHOLE PATIENT}\left\{0\right\}\text{HEALTH PROM}\left\{0,1\right\}\\ \text{COORD}\left\{0\right\}+\text{WHOLE PATIENT}\left\{0\right\}\text{SELF-MANAGE}\left\{0,1\right\} \end{array}$$

The findings may therefore be summarized as follows. The logical equations demonstrate that service improvements are perceived as happening differently across different services. Yet those who identified a state health office contribution to service improvement were different in some ways in the observed pattern of improvements (degree and kind) from those who did not. However, both groups did identify improvements under the different policy goals. When all possible missing cases are considered, a theoretical basis for concluding the two groups are different in terms of patterns of observed service improvement can also be demonstrated. There were also important theoretical differences between the two groups that did and did not observe improvements in relation to the sentinel policy goal of using evidence to better a service. The first group of this kind suggested multiple theoretical explanatory pathways variously involving big improvements in other policy goal areas. The second group suggested in two other equations offering explanatory pathways that reporting no improvement in this sentinel policy goal is accompanied by, and may be conditional on, no improvement in other service goal areas.

## Discussion and conclusions

In this study, the QCA analysis provided exploration of a method that with further development could 1) help better measure health service performance of policy goals in the context of interactions with the wider health system; 2) help gather stakeholder perceptions of service quality and improvement; 3) provide a new tool of analysis that allows multi-dimensional aspects of organizational performance, possibly even over time, to be captured and potentially triangulated with other data sources; 4) offer an approach to managing the interpretation of complexity-oriented outcome measures; 5) potentially help make benchmarking data more useful to policy-makers.

A strength of the QCA method is its capacity to describe all the different observed permutations of service improvements to help policy-makers see at a glance what kinds of perceived service improvements characterize what kinds of outcomes (e.g. in this study, those who can see a contribution from state health office actions and those who can’t). This focus in QCA upon describing an observed set can help deepen policy knowledge about possible relationships between state office efficacy and health service performance of policy goals.

A key benchmarking challenge for policy-makers is identifying what degree of each kind of improvement is appropriate. For example, if it is harder to achieve a modest improvement in one area because it relies on great improvement in another area, this is important information in realistic goal setting. The QCA method can help provide that information by identifying what combinations of what kinds and degrees of improvements can be observed or not observed in a health system. Thus, it can contradict simplistic cultural narratives such as ‘the system is not improving’ in ways that focus attention on the more nuanced truth that is about the complex ways in which different kinds of changes may be reliant on one another.

A concrete example of this interdependence is given in the QCA findings for those who saw at least some improvement in evidence-based service delivery. It appears that perceptions of improvement in this policy goal occur alongside, for example, big improvements in other areas such as whole-of-patient-care or health promotion or patient self-management. A policy priority that is about targeting this policy goal of creating a culture of using evidence would engage with understanding the barriers behind achieving change also in these other policy areas.

Accordingly, QCA could also help develop empirically based theory about how service improvements work in health systems. Can we generalize about what kinds of service improvements occur in concert, to what degree, in health systems? Very little is known about the answer to such questions about how the improving parts of a health system work (or do not work) together or how services operate in concert in a state health system. The QCA approach produces logical equations that can help describe such configurations of improvements and build better theory about health system operation. There is no doubt though, that it is a method that requires much more work and validation, including through traditional quantitative and qualitative methods, before the theory it suggests can be confidently translated into policy practice. It is also an approach that requires an effort of sustained learning from both the researcher and the research reader about how to re-conceptualize research methods in terms of ‘configurations’ of individual cases.

It could be argued that a key limitation of this study is that it did involve a random sample of services and not a theory-based purposeful selection of services and that the heterogeneity of services itself explains the lack of distinctive patterns of improvement (kind and degree). We do not believe there is a sufficient body of theory to make the selection of services involved. Further, the policy goals being measured are exceedingly broad such that it could be fairly assumed that practitioners and service managers interpreted them in ways appropriate to their services, as we encouraged them to do on the survey form and in telephone prompts. Heterogeneous results were obtained in this study for services with similar delivery modes and services with dissimilar delivery modes i.e. hospital units compared one to another were as heterogeneous as hospital units compared to community health centres. However, differences in the cultural attitudes of staff in those services or management achievement of change etc. might offer explanations of this heterogeneity.

This leads to another key limitation (and possibly strength) of the study: reliance on perceptions of improvement. It could also be argued that other data sources should have been used to establish the degree of improvement at the different services because stakeholder perceptions are not robust measures. Improvement may be occurring and not be perceived; it may not be occurring and be perceived as occurring. Willingness to acknowledge that improvement is occurring on a survey form is also shaped by different factors. Accordingly, a survey of perceptions does not offer empirical evidence that improvement is actually happening in a state health system or that state government actions are driving (or not driving) that improvement. However, we would argue that perceptions of service improvement are worth analyzing because they suggest the experience of service improvement and service staff cultures in which policy-makers operate. Policy-makers are strategic leaders who must also know about and shape such perceptions to try to achieve change. In this sense ‘perceptions’ are valid data for policy-makers. The fact that our study showed heterogeneity of perceptions provided the basis for refuting claims in this health system (which has some of the worst health outcomes in Australia [49,50]) that ‘no one believes improvement is happening’.

The study was able to show policy-makers that the story of perceptions of whole-of-system improvement is much more complex that such simplistic arguments (found for example in the media) suggest. We showed this in a context in which Tasmania, like many other Australian states, does not have sufficient data to properly assess the achievement of this range of broad policy goals across the state health system. The reason that we undertook exploration of adjunct methods such as QCA for this consultancy was that an early review of all relevant data sources for chronic disease, led by a senior epidemiologist, produced the conclusion that at this time the data collection infrastructure does not exist in a form that allows empirical assessment of the achievement of the eight policy goals driving state health service development for chronic disease. In a context in which stakeholder perceptions are seen in the benchmarking literature as legitimate adjunct data in understanding state health system performance, our study demonstrates how QCA might add value to understanding the complexities of service improvement, with all the limitations we describe.

A further possible weakness of the QCA method employed could be argued to be the fact that, unlike traditional statistical methods, the analysis does not deliver up a precise numerical value. However, we would point out that the method is designed to facilitate broad qualitative judgments in adjunct studies or be used in contexts where limited quantitative data are available, not act as a substitute for traditional quantitative analyses. In the case of stakeholder perceptions of service improvement of policy goals, these are only broad professional judgments that may not be accurately calibrated on finer scales than we have used for our QCA analysis.

Notwithstanding, the weaknesses of the QCA method need further exploration. In contexts where big-*N* traditional statistical methods are possible, it has clear limitations (although, of course, not all policy decisions need rely on traditional statistical reasoning). Further, QCA can create more complexity than it can solve. The explanatory pathways this study created may be plausible but they also suggest that the more complex the logical equations and their underlying constructs (here, policy goals), the more difficult they will be to empirically validate. This difficulty is, of course, often also encountered with quantitatively sophisticated approaches measuring complex phenomena. In short, the QCA method presents as a possibly valuable small-*N* adjunct method for benchmarking, rather than a singular solution to the challenges that beset a measurement-minded age. In the health service research field which has a strong history of embracing new methods, it will hopefully find a wider circle of researchers to pioneer its application in new areas such as benchmarking service performance.

## Appendix

### Technical appendix with key QCA concepts

The analysis was undertaken using Qualitative Comparative Analysis (QCA), a small-*N* method from the social sciences. Qualitative Comparative Analysis is different from either traditional quantitative analyses or traditional qualitative analyses. Traditional quantitative analyses involves numerical inputs and outputs and often involves probabilistic approaches to establishing whether an association between variables is unlikely to be due to chance. It focuses upon correlations across cases, not summarizing the combinations of conditions at work within single cases. Qualitative approaches typically involve a focus on analyzing language data in ways that lead to deeper understanding of complex phenomena i.e. the nature of community views about a particular policy issue. In contrast, the QCA method involves using a shorthand method for summarizing the combination of features that define individual cases i.e. its focus is upon economically describing the different configurations of features that define individual cases.

The QCA method can combine both quantitative and qualitative data (in this study perceptions of improvement in services and whether the state health office had a role in these) for individual cases. It considers how within-case conditions (e.g. in this study perceptions of service improvement) occur in relation to a particular outcome of interest (e.g. in this study whether the respondent could see a state health office contribution to service improvement). In QCA the focus is less on establishing what are the significant variables and more on establishing what features of all the cases considered are ‘necessary’ or ‘sufficient’ to a particular outcome of interest. A condition is necessary if all instances of the outcome occur with that condition. A condition is sufficient if all instances of the condition occur with the outcome [51-62]. For example, in relation to ‘sufficiency’, this method allows us to explore the question of whether one kind of perceived service improvement is always and in every case present on the survey forms of those who could see a state health office contribution i.e. it asks ‘Is there a particular kind of service improvement that, wherever it occurs in this data, is always found with the perception of a state health office contribution?’ Knowing the answer to such a question will help us learn whether policy-makers should target one kind of service improvement over another.

The QCA method could thus be described as a descriptive method: it focuses on describing the combinations of features that define individual cases. It can be easily seen why the QCA method is for situations where small numbers of cases are involved: too many cases and the combinations of case features involved are too many to summaries without losing a lot of information. In this study the focus is upon how combinations of perceived service improvement work one in relation to another in a context in which little is known about achievement of these different service improvement policy goals. Given that practitioners and service managers are informed observers of their services these data have potential value for understanding health service reform in complex contexts where policy-makers are trying to take a whole-of-system approach to chronic disease.

The main output of the software used to perform a QCA analysis is Boolean-based ‘logical equations’. This is a fairly simple system of notation used to summarise the combinations of case features. The logical equations produced can suggest whether particular features of cases are necessary or sufficient conditions for a specified outcome. However, in many contexts it may be that more complex causality is involved: the same outcome can be produced by different possibly causally related conditions such that conditions are neither necessary nor sufficient. [56,59-61,63-67] The number and complexity of analyses obtained for this study suggests this complex causality. When this happens, it suggests that the outcome can occur under different kinds of conditions: no single condition known to the study is critical to achieving the outcome. For example, what if willingness to see a state health contribution to service improvement occurred quite independently of any single observed service improvement? This would tell us that willingness to see a state health office contribution to service improvement might be caused by factors that lie outside the study.

Accordingly, QCS takes a focus upon complex causality precisely to help move qualitative understandings beyond simplistic approaches (‘No one really believes services are improving in the Tasmanian health system’) to more complex understandings conducive to better policy dialogue and targetted reform efforts (‘Most people can see improvement in at least one area but improvement isn’t perceived as happening uniformly—could it be that some kinds of improvements are necessary for others to happen?’)(‘If willingness to agree the state health office has made a contribution to improvement isn’t linked to a particular kind of service improvement actually being seen, could it be that the former is driven by other less rational factors?’).

An important feature of the QCA method is its treatment of missing cases. The software used by the QCA analyst allows consideration of every possible combination of the case features or variables entered by the researcher, not just those included in the observed set. These missing cases never observed in the study but theoretically possible are called ‘remainders’. ‘Simplifying assumptions’ are generated by the QCA software to reduce the number of possible combinations to brief summaries in the form of notation or ‘logical equations’ that describe what is possible theoretically, based on the observed number of cases. We could hypothesise that outside our sample of stakeholder perceptions there might be every possible combination (degree and nature) of improvement observed in services not part of the study. However, what if we could sample the entire universe of cases? What would still remain true once that entire universe of cases was compared to the small set of actual observations collected on our survey forms? The QCA method allows all these possible permutations of service improvement views to be considered and compared with known service improvement views to obtain a summary of minimized combinations or configurations—the logical equations [63].

Thus QCA can be described as a way of summarizing combinations of case features, whether those cases are observed in a dataset or whether they are theoretically possible based on what has been observed, already known or possible. This summarizing or ‘minimization’ of case detail involves a pairwise consideration of cases as described by Ragin, its principal proponent: ‘If two Boolean expressions differ in only one causal condition yet produce the same outcome, then the causal condition that distinguishes the two expressions can be considered irrelevant and can be removed to create a simpler, combined expression [51].’ (p.93) Thus in treating missing cases QCA does not involve inferences about what particular variables of cases are correlated but rather establishing what variable or case conditions are necessary and sufficient (or neither), given what is known about a limited number of cases (or what is theoretically possible).

The method has been well disseminated in many social sciences disciplines, with emerging studies in health services [63]. There are software programs in the public domain [64], as well as textbooks and summer schools on QCA methods.

In the present study, the ‘multi-value QCA’ or MVQCA approach is used and its presentation style [68]. The method is one of a number of variants of QCA which requires the software TOSMANA (developed by Lasse Cronqvist, Political Science, University of Trier, http://www.tosmana.net/). The MVQCA approach as articulated by Cronqvist, drawing on the work of Ragin, allows for consideration of different grades of responses that are treated as substantively different in the MVQCA algorithm:

## Competing interests

The authors declare that they have no competing interests.

## Authors’ contributions

EB conceptualized and wrote the first draft of the paper. BS provided major input on all the subsequent revisions of the paper, particularly as they relate to how to better describe the QCA method. All authors read and approved the final manuscript.

## Pre-publication history

The pre-publication history for this paper can be accessed here:

http://www.biomedcentral.com/1472-6963/12/343/prepub
